# Premature Translational Termination Products Are Rapidly Degraded Substrates for MHC Class I Presentation

**DOI:** 10.1371/journal.pone.0051968

**Published:** 2012-12-14

**Authors:** Joshua R. Lacsina, Odessa A. Marks, Xiongfei Liu, David W. Reid, Sujatha Jagannathan, Christopher V. Nicchitta

**Affiliations:** 1 Department of Pathology, Duke University School of Medicine, Durham, North Carolina, United States of America; 2 Department of Cell Biology, Duke University School of Medicine, Durham, North Carolina, United States of America; 3 Department of Molecular Biomedical Sciences, College of Veterinary Medicine, North Carolina State University, Raleigh, North Carolina, United States of America; 4 Department of Biochemistry, Duke University School of Medicine, Durham, North Carolina, United States of America; University of Nebraska Medical Center, United States of America

## Abstract

Nearly thirty percent of all newly synthesized polypeptides are targeted for rapid proteasome-mediated degradation. These rapidly degraded polypeptides (RDPs) are a source of antigenic substrates for the MHC class I presentation pathway, allowing for immunosurveillance of newly synthesized proteins by cytotoxic T lymphocytes. Despite the recognized role of RDPs in MHC I presentation, it remains unclear what molecular characteristics distinguish RDPs from their more stable counterparts. It has been proposed that premature translational termination products may constitute a form of RDP; indeed, in prokaryotes translational drop-off products are normal by-products of protein synthesis and are subsequently rapidly degraded. To study the cellular fate of premature termination products, we used the antibiotic puromycin as a means to experimentally manipulate prematurely terminated polypeptide production in human cells. At low concentrations, puromycin enhanced flux into rapidly degraded polypeptide pools, with small polypeptides being markedly more labile then high molecular weight puromycin adducts. Immunoprecipitation experiments using anti-puromycin antisera demonstrated that the majority of peptidyl-puromycins are rapidly degraded in a proteasome-dependent manner. Low concentrations of puromycin increased the recovery of cell surface MHC I-peptide complexes, indicating that prematurely terminated polypeptides can be processed for presentation via the MHC I pathway. In the continued presence of puromycin, however, MHC I export to the cell surface was inhibited, coincident with the accumulation of polyubiquitinated proteins. The time- and dose-dependent effects of puromycin suggest that the pool of peptidyl-puromycin adducts differ in their targeting to various proteolytic pathways that, in turn, differ in the efficiency with which they access the MHC I presentation machinery. These studies highlight the diversity of cellular proteolytic pathways necessary for the metabolism and immunosurveillance of prematurely terminated polypeptides that are, by their nature, highly heterogeneous.

## Introduction

Studies of eukaryotic protein turnover have revealed that nearly a third of all newly synthesized polypeptides are targeted for rapid degradation by the ubiquitin-proteasome system [1]. These rapidly degraded polypeptides (RDPs) have an average half-life of 10 minutes and comprise approximately 70% of proteasomal substrates [2]. Additionally, RDPs are a prominent source of antigenic peptides presented on major histocompatibility class I (MHC I) molecules [1], [3], [4]. A fraction of all protein synthesis appears to be directed to the RDP pool, even proteins that are metabolically stable and so it appears that newly synthesized polypeptides can be directed either to the pool of stable proteins, which display an average half-life of 1–2 days, or to the RDP pool [5], [6]. It is unclear, however, what molecular characteristics distinguish substrates directed to the stable protein pool versus the RDP pool.

Products of premature translational termination have previously been proposed to represent a source of RDPs [7], [8]. Support for this model comes from studies of *E. coli*, where it has been estimated that nearly 25% of translation initiation events result in the production of prematurely terminated translation products [9]–[11]. Consistent with these findings, peptidyl-tRNAs accumulate and are toxic to *E. coli* lacking functional peptidyl-tRNA hydrolase (Pth) [12], [13]. While there is no direct evidence for peptidyl-tRNA drop-off in eukaryotes, a number of experimental observations consistent with premature translational termination have been reported. First, premature termination would be predicted to cause ribosomal dissociation upstream of the termination codon, resulting in a relative decrease in ribosomal density towards the 3′ end of transcripts. In support of this model, studies of the distribution of ribosomes on mRNAs by both polysome microarrays [14] and ribosomal footprinting [15] indicate higher ribosomal density at the 5′ end of mRNAs. Second, specific sequences in the mRNA of Epstein-Barr Virus encoded nuclear antigen 1 (EBNA1) regulate the production of prematurely terminated EBNA1 polypeptides, which in turn serve as a source of MHC I peptides [16]. Finally, the presence of Pth homologs in eukaryotes [17], [18] suggests conservation of the mechanisms for premature translational termination and disposal of the resulting drop-off products.

To investigate the fate of translational drop-off products in human cells, we used the antibiotic puromycin. As a structural mimic of tyrosyl-tRNA, puromycin is covalently incorporated at the C-terminus of elongating nascent chains, leading to their dissociation from the ribosome as peptidyl-puromycin adducts [19]. While puromycin has been used previously to study the degradation of abnormal proteins (reviewed in [20]), those studies were conducted prior to the advent of membrane-permeable proteasome inhibitors, which enable the accurate quantitation of RDPs [21]. Following the development of puromycin-specific antibodies [22], puromycin has emerged as a useful tool to study the biology of defective ribosomal products (DRiPs) in a variety of cell types [23], [24]. In the present study, we employ both quantitative biochemical analysis and assays of antigen presentation to study the fate of puromycin-elicited premature translational termination products.

## Materials and Methods

### Materials

Cycloheximide (CHX), puromycin (puro) and the proteasome inhibitor MG132 were purchased from Sigma (St. Louis, MO). Mouse anti-K^b^ and corresponding isotype control antibodies were purchased from BD (Franklin Lakes, NJ). AlexaFluor 647 (AF647)-conjugated goat anti-mouse IgG, anti-green fluorescent protein (GFP) rabbit serum, and methionine/cysteine-deficient Dulbecco’s Modified Eagle Medium (Met/Cys- DMEM) were purchased from Invitrogen (Carlsbad, CA). The following reagents were also used: trichloroacetic acid (TCA, Mallinckrodt Chemicals, Phillipsburg, NJ), FK2 mouse anti-mono- and polyubiquitin conjugate (Millipore, Billerica, MA), and E7 mouse anti-β-tubulin (Developmental Studies Hybridoma Bank, University of Iowa, Iowa City, IA). Anti-puromycin rabbit serum was kindly provided by Peter Walter (UCSF, San Francisco, CA). The following were the kind gifts of Jon Yewdell (NIAID, Bethesda, MD): AF647-conjugated 25-D1.16, a monoclonal mouse antibody specific for the MHC class I-peptide complex K^b^-SIINFEKL [25], human embryonic kidney 293 cells stably expressing the mouse MHC class I allele, H-2K^b^ (293-K^b^) [26], and a plasmid containing NP-SIINFEKL-eGFP (NSe), composed of influenza nucleoprotein (NP) fused to the ovalbumin antigenic peptide SIINFEKL and enhanced green fluorescent protein (eGFP) [2]. NSe was subcloned into pcDNA6B (Invitrogen) for transfection-based expression. A reporter variant was generated containing eight additional tandem repeats of the SIINFEKL peptide inserted next to the existing SIINFEKL element in NSe, yielding the construct TRx9.

### Cell Culture

293-K^b^ cells were cultured in DMEM with 10% fetal bovine serum at 37°C and 5% CO_2_.

### Metabolic Radiolabeling and Pulse-chase

Radiolabeling and pulse-chase conditions to measure RDPs were adapted from [21]. Briefly, 293-K^b^ cells were resuspended at a concentration of 10^7^ cells/ml in methionine-deficient (Met/Cys-) DMEM with 1 mM glutamine, 1 mM sodium pyruvate and 25 mM HEPES. Cells were labeled with 300 µCi/ml ^35^S-methionine/cysteine (EasyTag Express Protein Labeling Mix, Perkin Elmer, Waltham, MA) at 37°C. To terminate labeling and precipitate polypeptides, TCA was added to a final concentration of 10% w/v and samples were incubated on ice for 10 min. Precipitates were washed twice in acetone, air-dried, resuspended in solubilization buffer (5% SDS, 0.5 M Tris) and heated at 95°C for 10 min to fully solubilize polypeptides. Radiolabeled polypeptides were measured by liquid scintillation counting or were separated on 10% Tricine SDS-PAGE gels [27]. Gels were dried and exposed to a PhosphorImager plate overnight at room temperature or to film at −80°C for 4 days. Custom Python scripts were written to quantify the sum of pixel intensities at each vertical position in an SDS-PAGE lane, with the sum plotted as a function of vertical position in the gel. To calculate the total signal in a specified region of a lane, the sum of pixel intensities was integrated over the given range.

For pulse-chase experiments, cells were radiolabeled as described above for 5 minutes. Pulse labeling was terminated by the addition of >10-fold excess ice-cold chase solution (Dulbecco’s phosphate buffered saline (DPBS) with 1% bovine serum albumin (BSA), 10 mM unlabeled methionine, 200 µM CHX) and placing the samples on ice. Cells were washed twice with chase solution, resuspended in chase media (Met/Cys- DMEM with 10 mM methionine and 200 µM CHX) and incubated at 37°C; the chase was terminated at specific time points by the addition of TCA as described above.

### Denaturing Immunoprecipitation

Solubilized TCA precipitates from radiolabeled cells were used at a concentration of 4×10^4^ cell equivalents per 10 µl of solubilization buffer. For each immunoprecipitation (IP) reaction, 10 µl of solubilized TCA precipitate was diluted into 990 ml of IP buffer (1% Triton X-100, 25 mM HEPES, 150 mM NaCl, 1 mM EDTA) and precleared with 15 µl of Pansorbin cells (EMD, Gibbstown, NJ) for 30 minutes at room temperature. To precipitate peptidyl-puromycins, 1 µl of anti-puromycin serum was incubated with the precleared lysate for 1 hour at room temperature with gentle mixing. Immune complexes were captured by adding 15 µl of a 50% slurry of Pierce protein A/G agarose beads (Thermo Fisher, Rockford, IL) and incubating for 1 hour at room temperature with gentle mixing. Beads were washed four times with 1 ml IP buffer, then mixed with 22 µl sample buffer (300 mM Tris, pH 6.8, 36% glycerol, 10% SDS, 0.012% bromophenol blue) with 50 mM dithiothreitol (DTT) and heated to 95°C for 5 minutes. Beads were pelleted and the supernatants were used for scintillation counting and tricine SDS-PAGE.

### Flow Cytometry

To measure cell surface K^b^ complexes, 293-K^b^ cells were harvested and stained with 0.5 µg of either isotype control or anti-K^b^ antibody in 100 µl of FACS buffer (DPBS with 1% BSA and 0.02% sodium azide) on ice for 45 minutes. Cells were washed twice with FACS buffer, and then stained with 1 µg of AF647-goat anti-mouse IgG in 100 µl of FACS buffer on ice for 45 minutes. Cells were washed twice more with FACS buffer, then resuspended in 300 µl FACS buffer with 2 µg/ml propidium iodide (PI, Sigma-Aldrich, St. Louis, MO) on ice for 30 minutes. A similar procedure was used to measure cell surface K^b^-SIINFEKL complexes using the AF647-conjugated 25-D1.16 monoclonal antibody (1∶500 dilution) except for the exclusion of a secondary antibody staining step. Samples were analyzed immediately using an LSRII flow cytometer (BD Biosciences, San Jose, CA). PI-positive cells were excluded from analyses of cell surface antibody staining. For each fluorescence channel, the minimum and maximum values of geometric mean fluorescence intensity (MFI) were standardized between trials. All flow cytometry data were analyzed using FlowJo version 8.6.1 (Treestar, Ashland, OR).

### MHC Class I Peptide Stripping and Recovery

Twenty-four hours prior to reporter plasmid transfection, 293-K^b^ cells were seeded into 10-cm plates at a density of 10^6^ cells/plate. For transfection, 54 µg of polyethylenimine (PEI, 25 kDa, linear, Polysciences, Warrington, PA) was complexed with 18 µg of reporter plasmid DNA in Opti-MEM (Invitrogen) and incubated with cells for 7 hours. Media containing PEI-DNA complexes was exchanged for fresh, prewarmed media, and transfected cells were incubated for an additional 17 hours. Cells were then harvested and stripped of MHC I peptides as described in [28]. Briefly, cell pellets were resuspended in peptide stripping buffer (0.131 M citric acid, 0.66 M Na_2_HPO_4_, 1% BSA, pH 3) and incubated on ice for 2 minutes. The pH was neutralized to 7.4 and the cells were resuspended at a concentration of 10^5^ cells/ml in standard media, then seeded into a 12-well plate with varying concentrations of protein synthesis inhibitors. Cells were incubated for up to 4 hours at 37°C to allow for the recovery of cell surface MHC class I-peptide complexes. A subset of samples was treated with 5 µM SIINFEKL peptide for the final 30 minutes of the recovery period. The cells were harvested at the indicated time points, stained for cell surface K^b^ and K^b^-SIINFEKL complexes, and analyzed by flow cytometry as described above.

### Western Blotting

293-K^b^ cells were harvested and lysed on ice in IP buffer with 1 mM phenylmethylsulfonyl fluoride (PMSF) for 10 minutes. Lysates were clarified by centrifugation at 20000×*g* for 10 minutes at 4°C. Lysates were mixed 1∶1 with sample buffer and 20 mM DTT, heated to 95°C for 5 minutes, and separated by SDS-PAGE on standard 10% Laemmli gels, using 3×10^5^ cell equivalents/lane. Proteins were transferred via overnight wet transfer onto a polyvinylidene fluoride (PVDF) membrane and probed for β-tubulin (E7) and mono- and polyubiquitinated proteins (FK2). After incubation with HRP-conjugated secondary antibodies, proteins were detected using the SuperSignal West Pico Chemiluminescent Substrate (Thermo Scientific) according to the manufacturer’s instructions.

## Results

### Development and Characterization of a Model System to Study the Products of Premature Translational Termination

We sought to establish puromycin as a convenient tool to produce and track the fate of premature translational termination products in cells. To identify a puromycin concentration range that promotes the production of prematurely terminated polypeptides while maintaining a substantial level of protein synthesis, 293-K^b^ cells were radiolabeled with [^35^S]-Met in the presence of varying concentrations of puromycin and radioisotope incorporation was then quantified as a measure of total cellular protein synthesis. For comparison, we used cycloheximide (CHX), which arrests elongating ribosomes without prematurely discharging the nascent chain [19].

**Figure 1:**
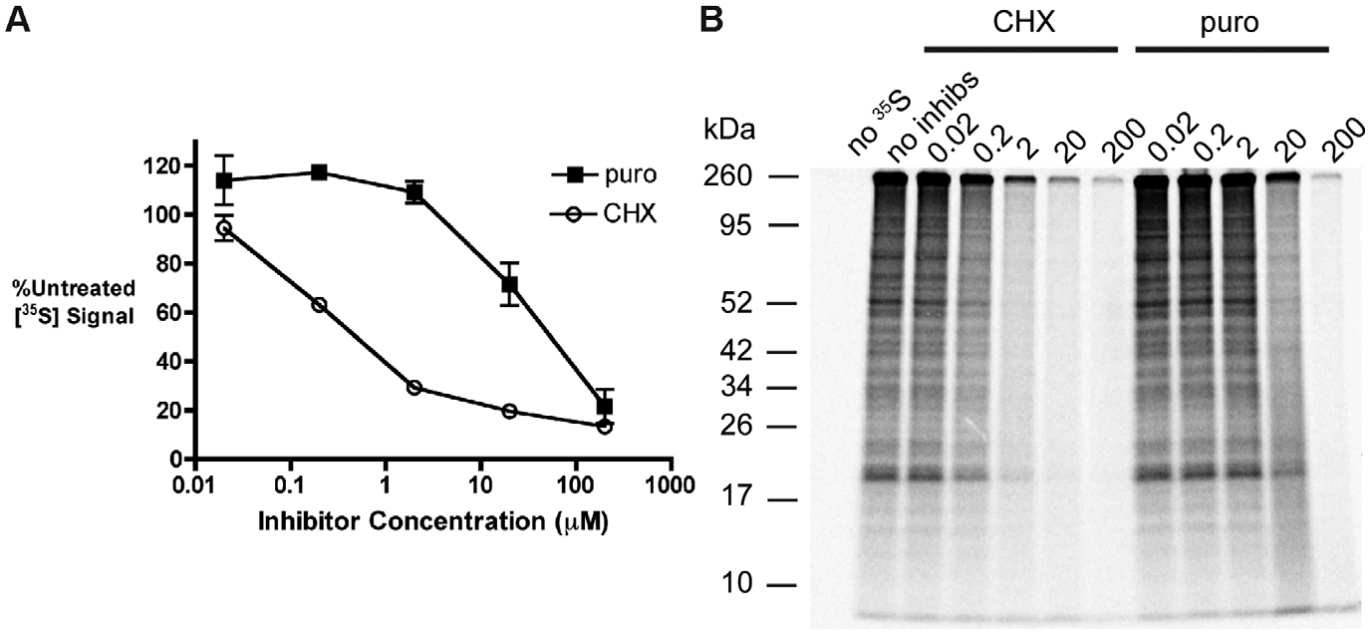
Contrasting effects of cycloheximide and puromycin on the profile of newly synthesized polypeptides. *A.* 293-K^b^ cells were radiolabeled with [^35^S]-Met for 10 minutes in the presence of varying concentrations (µM) of CHX and puro, followed by precipitation of polypeptides with trichloroacetic acid (TCA). TCA precipitates were solubilized and radiolabel incorporation was measured by liquid scintillation counting. [^35^S] signal was normalized to untreated controls (*n* = 3; mean ± s.e.m.) *B*. Separation of TCA precipitates from samples in *A* by tricine SDS-PAGE in 10% gels. Gels were dried and exposed to a PhosphorImager plate overnight. Results are representative of three independent experiments.

The data in Fig. 1A illustrate the protein synthesis dose inhibition profiles for puromycin and CHX. CHX elicited a biphasic inhibition of [^35^S] incorporation, with ∼70% inhibition over a 2 log order increase in concentration and ∼20% inhibition over an additional 2 log order increase. In contrast, treatment with 0.2 to 2 µM puromycin resulted in a small but significant increase in [^35^S] labeling compared to untreated controls (*p* = 0.01 and *p* = 0.07 at 0.2 and 2 µM, respectively), with ∼90% inhibition occurring over the remaining 2 log order increase in concentration. The stimulation of protein synthesis observed at low puromycin concentrations is consistent with previous reports demonstrating that moderate accumulation of misfolded proteins induces a small increase in protein synthesis [29]. These results demonstrate that inducing premature termination with low concentrations of puromycin allows the majority of total protein synthesis to be maintained (20 µM) or causes it to slightly increase (0.2–2 µM).

To examine the molecular characteristics of the puromycin-elicited drop-off products, lysates from cells radiolabeled with varying concentrations of puromycin or CHX were separated by SDS-PAGE and [^35^S]-labeled proteins were first visualized by phosphorimaging (Fig. 1B). Increasing concentrations of CHX resulted in progressive decreases in [^35^S] signal that uniformly affected polypeptides of all molecular weights. In contrast, the profile of newly synthesized polypeptides was relatively unaffected by puromycin concentrations from 0.02 to 2 µM, consistent with the [^35^S] incorporation data (Fig. 1A). This profile shifted markedly at 20 µM puromycin, where radiolabel incorporation into the prominent bands was reduced coincident with an increase in background radioactivity throughout the lane, consistent with the appearance of highly heterogeneous, radiolabeled species. There was also an increase in [^35^S] signal from unresolved polypeptides at the dye front. At 200 µM puromycin, there was a nearly complete loss of [^35^S]-labeled polypeptides.

**Figure 2:**
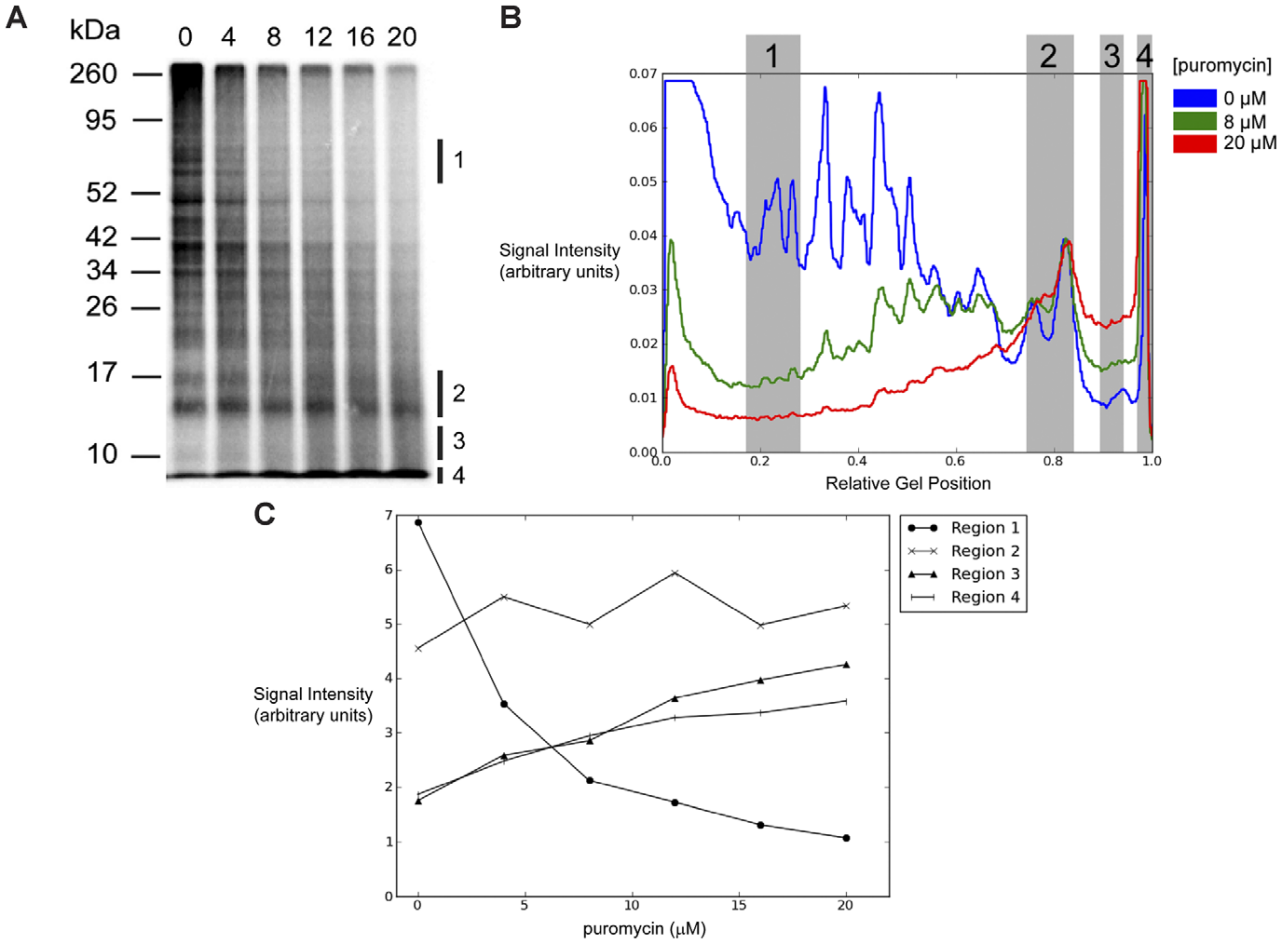
Puromycin stimulates the production of truncated polypeptides in a dose-dependent manner. *A.* 293-K^b^ cells were radiolabeled with [^35^S]-Met for 10 minutes in the presence of a linear range of puromycin concentrations from 0 to 20 µM. Radiolabeled polypeptides were visualized as described for Fig. 1B. In the later panels, we analyze the [^35^S] signals from the four regions indicated to the right of the gel. *B*. PhosphorImager signal intensities (arbitrary units) from selected lanes in *A*. The left side of the graph corresponds to the top of the gel while the right side of the graph corresponds to the bottom of the gel at the dye front. The highlighted regions correspond to the parts of the gel indicated in *A*. *C*. The effects of puromycin concentration on [^35^S] signal for each of the highlighted gel regions in *A* and *B*. Results are representative of three independent experiments.

To further characterize the heterogeneous radiolabeled protein fractions present in puromycin-treated cells, cells were radiolabeled with [^35^S]-Met over a linear range of puromycin concentrations from 0 to 20 µM and the labeled polypeptide composition was analyzed by SDS-PAGE (Fig. 2A). The profiles of radiolabeled polypeptides showed a puromycin-dependent shift in the peak intensity of [^35^S] signals from higher to lower molecular weight polypeptides. There was also a puromycin-dependent increase in signal for unresolved low molecular weight polypeptides at the dye front. The gel data were plotted in Fig. 2B to visualize lane intensity profiles. Additionally, the effects of varying puromycin concentration on [^35^S] signal were determined for polypeptides of different molecular weights (Regions 1–4 throughout Fig. 2, plotted in Fig. 2C). For the gel regions analyzed, puromycin treatment resulted both in the progressive loss of polypeptides from ∼60–85 kDa in size (Region 1) and in the accumulation of polypeptides ∼10–12 kDa (Region 3) and <10 kDa (Region 4) in size, reflecting increased production of truncated polypeptides. In summary, treatment with 20 µM puromycin markedly enhanced the production of premature termination products while maintaining a substantial fraction (∼70%) of total protein synthesis (Fig. 1A). These experimental conditions were used in the studies described below to study the cellular fate of prematurely terminated polypeptides.

### The Products of Premature Translational Termination are Rapidly Degraded

**Figure 3:**
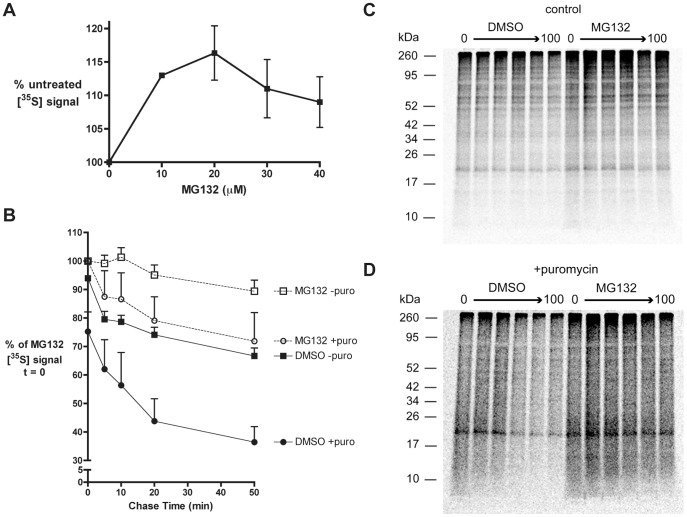
Treatment with puromycin increases the fraction of rapidly degraded polypeptides. *A.* 293-K^b^ cells were labeled with [^35^S]-Met for 10 minutes in the presence of 0 to 40 µM MG132. [^35^S] incorporation was measured as in Fig. 1A and normalized to controls without proteasome inhibitor (*n* = 3; mean ± s.e.m.) *B.* 293-K^b^ cells were pulse labeled with [^35^S]-Met +/−20 µM puro and +/−20 µM MG132 for 5 minutes, then chased from 0 to 50 minutes in the presence of excess cold methionine, CHX and +/−20 µM MG132. DMSO is a solvent control for MG132. The chase was terminated at the indicated time points by the addition of TCA to cell suspensions to precipitate polypeptides. TCA precipitates were solubilized and [^35^S] was measured by liquid scintillation counting (*n* ≥4; mean ± s.e.m.) *C* and *D*. Solubilized TCA precipitates from cells radiolabeled in the absence (*C*) or presence (*D*) of 20 µM puro were separated by tricine SDS-PAGE on 10% gels. Gels were dried and exposed to a PhosphorImager plate overnight. Note that for *D*, the darkness of the image has been enhanced in order to see the contrast in degradation rates between *C* and *D* more clearly.

Treating cells with the proteasome inhibitor MG132 has been shown to rescue RDPs from degradation [1]. To determine the MG132 concentration that leads to maximal RDP recovery, 293-K^b^ cells were radiolabeled with [^35^S]-Met for 10 minutes in the presence of varying concentrations of MG132 (Fig. 3A). Maximal recovery of newly synthesized polypeptide was achieved at 20 µM MG132, which was chosen as the working concentration of proteasome inhibitor for the remaining experiments. At MG132 concentrations greater than 20 µM, a trend towards decreased recovery of radiolabeled protein was observed. We speculate that this decrease reflects indirect inhibition of protein synthesis by high concentrations of MG132, as has been reported previously following prolonged treatments with proteasome inhibitors [1].

To assess the stability of premature termination products, metabolic pulse-chase experiments were performed to measure the fraction of RDPs [21]. Cells were pulse-labeled for 5 min in the presence or absence of 20 µM puromycin, then chased for up to 50 minutes without puromycin in the presence or absence of MG132 (Fig. 3B). In control cells without puromycin, 20% of the radiolabeled proteins were degraded by the proteasome 5 minutes into the chase, with a total loss of 25% of the radiolabel by the end of the chase relative to MG132-treated controls. These results are consistent with previously reported measures of the fraction of rapidly degraded polypeptides in mammalian cells [2], [26]. In contrast, by the beginning of the chase in puromycin-treated cells, 25% of the radiolabeled polypeptides had already been lost in a proteasome-dependent manner, with continued rapid loss of radiolabel up to 20 minutes into the chase. By the end of the 50 min chase in puromycin-treated cells, 49% of the radiolabeled polypeptides were degraded relative to MG132-treated controls. The increase in protein degradation following puromycin treatment indicated that the premature termination products were rapidly degraded. Interestingly, there was a trend towards increased degradation in cells treated with both puromycin and MG132. Comparing MG132-treated cells in the presence or absence of puromycin, there was an initial rapid drop in signal for puromycin-treated cells, with the eventual loss of 20% of the radiolabel by the end of the chase. These results indicate that a fraction of prematurely terminated polypeptides are degraded via an MG132-resistant mechanism.

To examine changes in the polypeptide profile under the different pulse-chase conditions, lysates from cells treated as described above were separated by SDS-PAGE and radiolabeled polypeptides were visualized by phosphorimaging. In control cells pulse-labeled without puromycin (Fig. 3C), there was a modest recovery of [^35^S] signal following MG132 treatment. For cells pulse-labeled with puromycin (Fig. 3D), MG132 treatment rescued [^35^S] signal for polypeptides of all sizes. In both the presence and absence of proteasome inhibitor, there was a marked loss in low molecular weight polypeptides for puromycin-treated cells over the course of the chase. These pulse-chase studies strongly suggest that prematurely terminated polypeptides induced by puromycin treatment are rapidly degraded.

**Figure 4:**
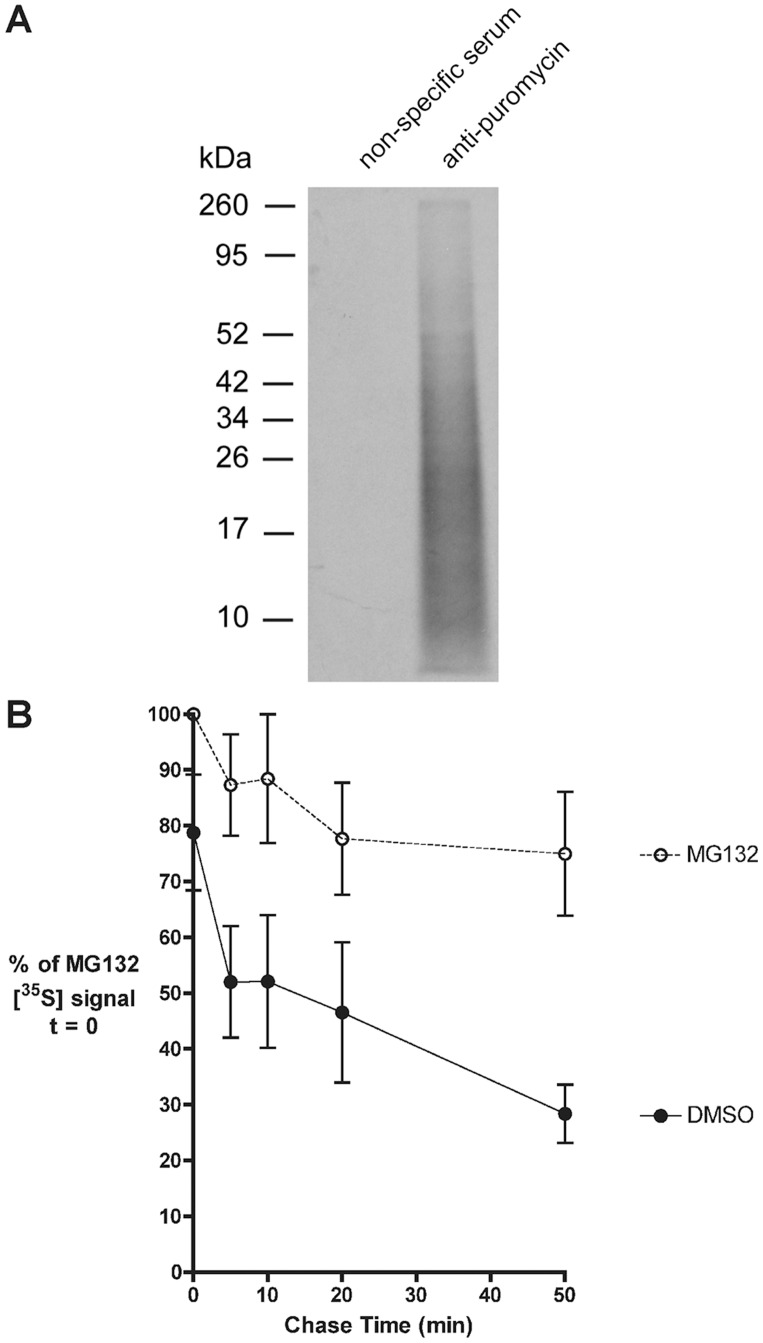
Rapid degradation of premature translational termination products. *A.* Denaturing immunoprecipitation of peptidyl-puromycins. 293-K^b^ cells were radiolabeled for 30 minutes with [^35^S]-Met, 20 µM puromycin, and 20 µM MG132. Polypeptides were precipitated with TCA, solubilized, then subjected to a denaturing immunoprecipitation using either non-specific rabbit serum (negative IP control) or anti-puromycin serum. Results are representative of three independent experiments. *B.* 293-K^b^ cells were pulse labeled with [^35^S]-Met and 20 µM puro and chased as described in Fig. 3B. Solubilized TCA precipitates were subjected to denaturing immunoprecipitation using anti-puromycin serum. [^35^S] in the anti-puromycin immunoprecipitates was measured by liquid scintillation counting (*n* = 4; mean ± s.e.m.).

To specifically analyze prematurely terminated polypeptides, denaturing immunoprecipitation (IP) experiments were performed using anti-puromycin antisera to capture the peptidyl-puromycin termination products [22]. First, the profile of immunoprecipitated peptidyl-puromycins was examined by radiolabeling cells with [^35^S]-Met in the presence of 20 µM puromycin (Fig. 4A). While non-specific rabbit sera precipitated no appreciable signal, anti-puromycin sera precipitated a smear with a signal peak of 19 kDa. This result demonstrates that the low molecular weight polypeptides that increase in intensity with puromycin treatment are peptidyl-puromycins.

After establishing conditions for a quantitative anti-puromycin IP, the degradation of peptidyl-puromycins was characterized by metabolic pulse-chase analysis. Cells were pulse-labeled for 5 min with [^35^S]-Met and 20 µM puromycin, then chased for up to 50 minutes in the presence or absence of MG132. Denaturing anti-puromycin IPs were performed on all samples to recover and measure peptidyl-puromycins by liquid scintillation counting (Fig. 4B). In the absence of MG132, 21% of the peptidyl-puromycins were degraded by the start of the chase. An additional 30% was degraded during the first 5 minutes of the chase, followed by a more gradual degradation profile. By the end of the chase, 62% of the peptidyl-puromycin signal was lost relative to MG132-treated cells. These results confirm that peptidyl-puromycins are subject to rapid degradation, with a nearly 2.5-fold higher fraction of rapidly degraded polypeptides compared to total protein from non-puromycin treated controls (Fig. 3B). Notably, in MG132-treated cells, 20% of the immunoprecipitated peptidyl-puromycin signal was lost by the end of the chase (Fig. 4B). This result mirrored the degradation profile of total protein from cells treated with both puromycin and MG132 (Fig. 3B), providing additional evidence for MG132-sensitive and -insensitive peptidyl-puromycin degradation pathways.

### Effects of Stimulating Premature Translational Termination on the Export of MHC Class I-peptide Complexes

**Figure 5:**
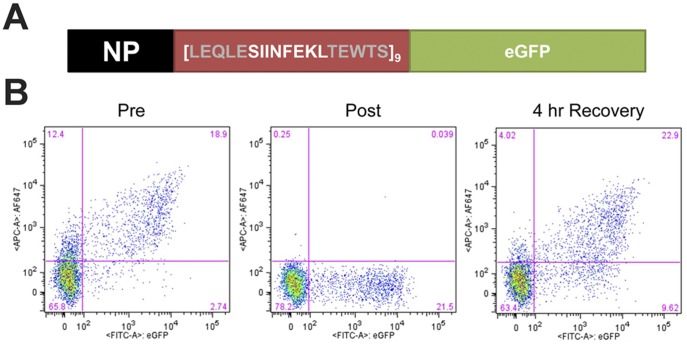
MHC class I-peptide complex recovery assay using a fluorescent reporter encoding antigenic peptides. *A.* Schematic of the modified NP-SIINFEKL-eGFP reporter (adapted from [2]) containing eight additional tandem repeats of SIINFEKL (nine total) and its five flanking amino acids from the native ovalbumin sequence, NP-[SIINFEKL]_9_-eGFP (Tandem Repeat x9 or TRx9). *B.* Validation of MHC I peptide stripping and recovery in TRx9-expressing cells. Biexponential scatter plots show single cell profiles of the mean fluorescence intensity (MFI) for eGFP on the *x*-axis and 25-D1.16 on the *y*-axis. Plots show fluorescence profiles immediately pre- (*left*) and post- (*middle*) peptide stripping, and after a 4 hour recovery (*right*).

Since RDPs are a source of peptides presented on MHC class I molecules [1], [3], [4], puromycin would be expected to increase the net production of RDPs, and thereby stimulate peptide presentation via the MHC class I pathway. This hypothesis was tested by measuring the recovery of cell surface MHC I-peptide complexes following acid stripping of MHC I-bound peptides. For these experiments, HEK293 cells stably expressing the murine MHC class I allele H-2 K^b^ (293-K^b^ cells) were used [26]. Cells were transfected with a reporter plasmid encoding a fusion protein of influenza nucleoprotein (NP), a 9-mer repeat of the antigenic peptide SIINFEKL (with flanking sequences for efficient processing), and eGFP (adapted from [2]) (Fig. 5A). Expression of the functional reporter protein produces fluorescence from the C-terminal eGFP tag. Degradation and processing of the reporter protein liberates antigenic SIINFEKL peptides from the internal tandem repeat element. These SIINFEKL peptides can be loaded onto K^b^ molecules in the endoplasmic reticulum (ER) and presented at the cell surface as K^b^-SIINFEKL MHC class I-peptide complexes. Brief treatment of cells with an acidic buffer efficiently dissociated cell surface K^b^-SIINFEKL complexes, with partial recovery after a 4 hr incubation at 37°C (Fig. 5B).

**Figure 6:**
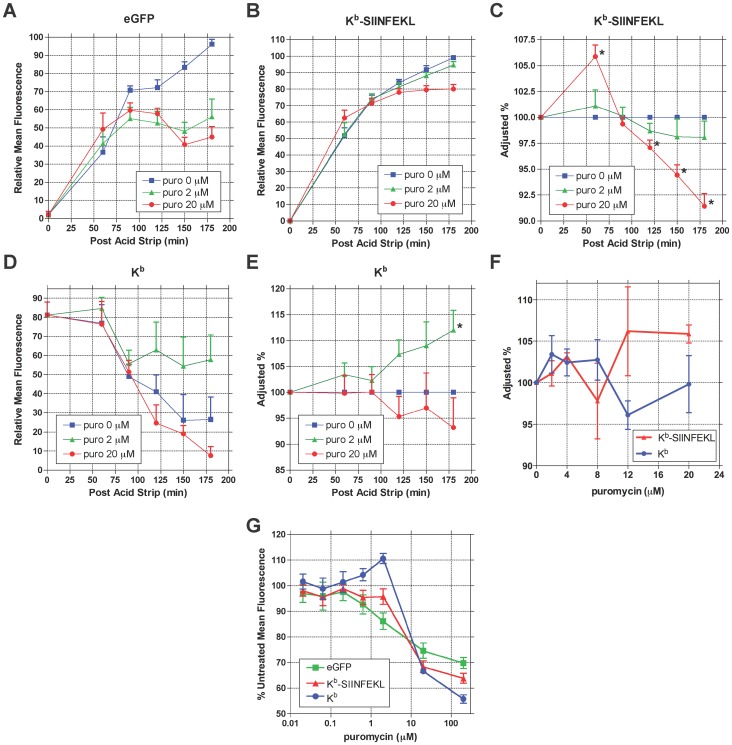
Effects of puromycin on the recovery of cell surface MHC class I-peptide complexes. *A*–*E*. 293-K^b^ cells expressing the TRx9 reporter were stripped of cell surface MHC I peptides as in Fig. 5. Recovery of cell surface MHC class I-peptide complexes was conducted in the presence of varying concentrations of puro from 0 to 180 minutes. Flow cytometry was used to measure reporter eGFP fluorescence (*A*), as well as cell surface K^b^-SIINFEKL complexes (*B*) and total cell surface K^b^ (*D*). To quantitate differences in the kinetics of MHC class I-peptide complex recovery, we normalized the MFI values of puro-treated cells to untreated cells for K^b^-SIINFEKL (*C*) and total K^b^ (*E*) as described in [4] (*n* = 5; mean ± s.e.m.) For *C* and *E*, * *p*<0.05 compared to untreated samples. *F.* MHC class I-peptide complex recovery after 1 hour in the presence of varying concentrations of puromycin. MFI values are normalized to untreated cells (*n* = 3; mean ± s.e.m.) *G*. MHC class I-peptide complex recovery after 4 hours in the presence of varying concentrations of puromycin. MFI values are normalized to untreated cells (*n* = 3; mean ± s.e.m.).

To examine the effects of puromycin on MHC I-peptide complex recovery, reporter-expressing cells were stripped of MHC I peptides and allowed to recover in the presence of varying concentrations of puromycin from 60 to 180 minutes. Sixty minutes was chosen as the initial time point as this is the approximate timeframe for protein degradation, peptide loading, and K^b^-SIINFEKL export to the cell surface [4]. Flow cytometry was used to measure functional reporter protein (eGFP fluorescence) as well as cell surface K^b^ and K^b^-SIINFEKL complexes. Puromycin treatment led to a decrease in eGFP fluorescence after a 90 min lag (Fig. 6A), consistent with the production of non-fluorescent, truncated reporter proteins. To more clearly ascertain the effects of puromycin on the cell surface expression of K^b^-SIINFEKL complexes, the raw data (Fig. 6B) for the puromycin-treated cells was normalized to control (untreated) cells (Fig. 6C) as described in [4]. While 2 µM puromycin caused no significant change in the recovery of cell surface K^b^-SIINFEKL complexes, treatment with 20 µM puromycin elicited an increase in K^b^-SIINFEKL levels, followed by a constant decline in the rate of K^b^-SIINFEKL presentation. These findings suggest that the initial burst of puromycin-induced drop-off products caused an increase in MHC I presentation, but that sustained production of truncated polypeptides inhibits the flux of the reporter gene epitope into the MHC I presentation pathway.

To generalize these findings to MHC I-peptide complexes other than K^b^-SIINFEKL, a similar experiment was conducted to measure total cell surface K^b^ recovery in the presence of varying concentrations of puromycin (Fig. 6D). Data from puromycin-treated cells were normalized to the untreated samples, as before (Fig. 6E). After acid stripping, cells displayed a time-dependent loss of cell surface K^b^ molecules (Fig. 6D). Because MHC I molecules lacking bound peptide show decreased cell surface stability [30], the loss of cell surface K^b^ after acid stripping reflects the shedding, unfolding, and internalization of destabilized K^b^ molecules [4]. Puromycin treatment had no discernible effect on K^b^ surface presentation during the first 90 minutes of recovery. Treatment with 2 µM puromycin led to accelerated delivery of K^b^ to the cell surface from 120 to 180 minutes, a trend reaching statistical significance at the 180 minute time point (Fig. 6E). Cells treated with 20 µM puromycin showed a trend towards impaired K^b^ surface presentation from 120 minutes onwards, though this did not reach statistical significance. The K^b^ recovery studies indicate that translational drop-off products induced by low concentrations of puromycin stimulate MHC class I presentation, whereas at higher concentrations, puromycin treatment suppresses the rate of MHC class I presentation. Puromycin concentrations less than 20 µM had no significant effect on either cell surface K^b^ or K^b^-SIINFEKL recovery after one hour (Fig. 6F). Thus, lower concentrations of puromycin do not stimulate K^b^ or K^b^-SIINFEKL presentation above what is seen with 20 µM puromycin. We speculate that the contrasting effects of puromycin on K^b^-SIINFEKL compared to K^b^ recovery can be attributed to the differences between the action of puromycin on the TRx9 reporter versus the global population of precursors for all K^b^-binding peptides.

A consistent observation in the analysis of cell surface presentation of K^b^-SIINFEKL and K^b^ is that higher concentrations of puromycin elicited a decrease in the rate of cell surface MHC class I presentation. To extend these observations, a similar acid stripping/recovery experiment was performed in the presence of increasing concentrations of puromycin. After 240 minutes of recovery, eGFP, K^b^-SIINFEKL, and K^b^ were measured by flow cytometry (Fig. 6G). Puromycin caused an initial dose-dependent enhancement in cell surface K^b^ levels (*p* = 0.03 for 2 µM), while the recovery of K^b^-SIINFEKL complexes was not significantly affected over this concentration range. The recovery of both total K^b^ and K^b^-SIINFEKL dropped precipitously at 20 µM puromycin. These studies suggest that a low frequency of premature termination events stimulates flux through the MHC I pathway, while higher production of prematurely terminated polypeptides inhibits MHC I presentation.

**Figure 7:**
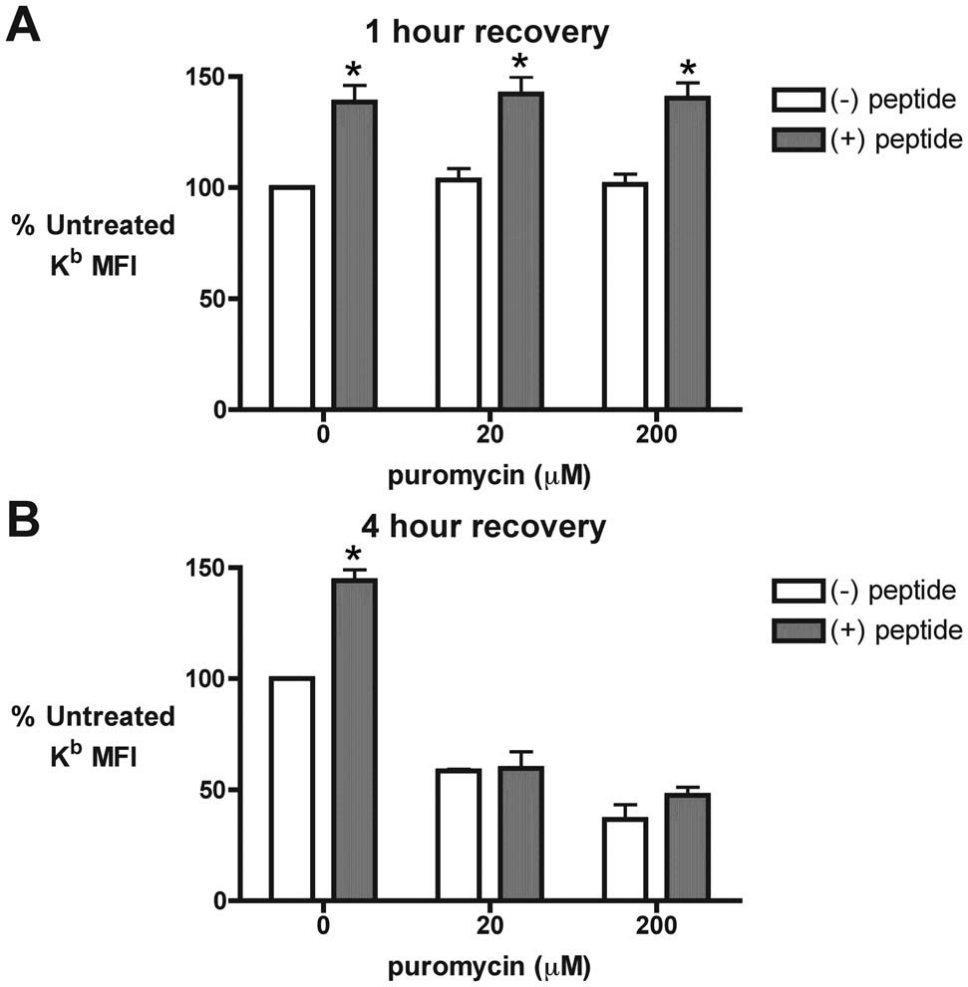
Time-dependent inhibition of MHC class I pathway function following puromycin treatment. *A* and *B.* 293-K^b^ cells were stripped of cell surface MHC I peptides as in Fig. 5. Recovery of cell surface K^b^ was conducted in the presence of varying concentrations of puro for one (*A*) and four (*B*) hours. During the final 30 minutes of the recovery, cells were treated with either distilled water ((−) peptide) or 5 µM SIINFEKL peptide ((+) peptide) to promote the export of K^b^ to the cell surface [31]. Flow cytometry was used to measure total cell surface K^b^ and the MFI was normalized to untreated cells in the absence of exogenous SIINFEKL peptide (*n* = 3; mean ± s.e.m.; * *p*<0.05 for (−) peptide vs. (+) peptide).

**Figure 8:**
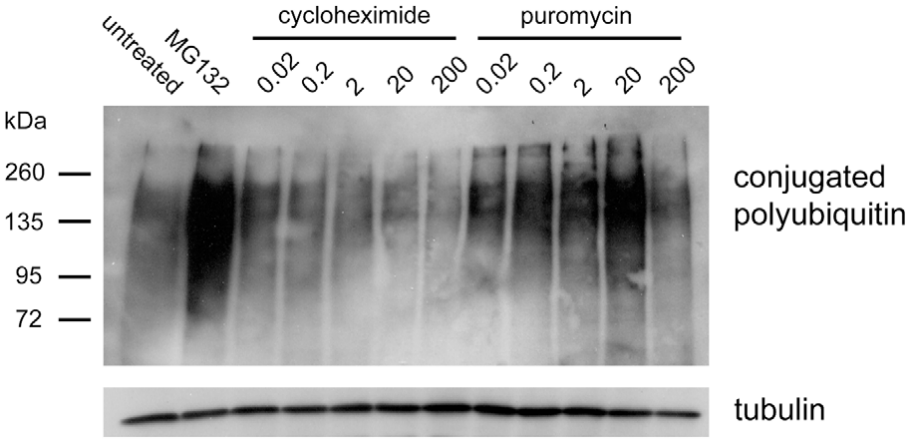
Puromycin treatment leads to increased levels of polyubiquitinated proteins. 293-K^b^ cells were treated for 4 hours with media alone, 20 µM MG132, or the indicated concentrations of cycloheximide and puromycin (µM). Lysates were subjected to Western blotting with FK2 (*upper panel*), a monoclonal antibody specific for mono- and polyubiquitinated protein conjugates. Beta-tubulin was probed as a loading control (*lower panel*). Results are representative of three independent experiments.

The inhibitory effects of puromycin on MHC I presentation were consistently observed to be more pronounced at later time points. To assess the functionality of the MHC I pathway machinery at different time points during puromycin treatment, cells were acid stripped and allowed to recover in the presence of puromycin for one or four hours. During the last 30 minutes of recovery, cells were treated with exogenous SIINFEKL peptide, which is transported in a retrograde fashion to the ER [31]. There the exogenous SIINFEKL peptides complex with empty K^b^ molecules and stimulate their export to the cell surface. However, in the absence of a functional MHC I pathway, adding SIINFEKL peptide to cells has no effect on cell surface MHC I levels. After 1 hour of recovery, treatment with SIINFEKL peptide increased cell surface K^b^ expression in both puromycin-treated and untreated cells (Fig. 7A). In contrast, after 4 hours of recovery, puromycin-treated cells showed no significant increase in cell surface K^b^ when incubated with SIINFEKL peptide, while untreated cells did not display this inhibition (Fig. 7B). These findings suggest that sustained treatment with puromycin impairs the function of the MHC I presentation machinery, leading to the marked inhibition of MHC I recovery seen at later time points. We postulated that the latter effect was partially due to saturation of the proteasome with premature termination products. Consistent with this interpretation, treating cells with 0.02–20 µM puromycin resulted in a dose-dependent accumulation of polyubiquitinated proteins (Fig. 8). This increase was not observed following treatment with 200 µM puromycin, which produces very short premature termination products. Furthermore, treatment with CHX, which prevents the drop-off of prematurely terminated polypeptides, caused a dose-dependent decrease in polyubiquitinated proteins, as observed previously [1]. These results highlight a correlation between increased production of prematurely terminated polypeptides, proteasomal inhibition, and impaired MHC I presentation.

## Discussion

These studies investigated the fate of premature translational termination products, which have been proposed to be a source for rapidly degraded polypeptides (RDPs) [7], [8]. The antibiotic puromycin was used to stimulate the production of truncated peptidyl-puromycins (Figs. 2 and 4A), which served as model prematurely terminated polypeptides. Puromycin treatment doubled the proteasome-sensitive, rapidly degraded polypeptide fraction in cells (Fig. 3). This enhanced degradation predominantly affected lower molecular weight polypeptides, including a fraction whose degradation was resistant to MG132. These unstable, small polypeptides were identified as premature termination products, an assertion supported by our immunoprecipitation studies demonstrating the peptidyl-puromycin profile (Fig. 4A) and their rapid degradation (Fig. 4B). A fraction of these peptidyl-puromycins was found to be degraded in an MG132-resistant manner. We conclude that peptidyl-puromycins are rapidly degraded polypeptides, supporting the hypothesis that premature translational termination products compose a general subclass of RDPs.

While the truncated polypeptides produced in our system are likely to be highly defective, both in conformation and function, the molecular signals that target peptidyl-puromycins for rapid degradation are unknown. Many of these peptidyl-puromycins are likely to be unfolded, bearing exposed hydrophobic patches that serve as degradation signals [32]. Targeting of peptidyl-puromycins for rapid degradation may be coupled to signals induced by ribosomal stalling, such as the no-go decay pathway. In support of this, the no-go decay factors Dom34 and Hbs1 were found to promote peptidyl-tRNA drop-off in response to stalling of the ribosome in an *in vitro* reconstituted translation system [33]. A related question is the extent to which peptidyl-puromycin degradation depends on polyubiquitination. We note that while the accumulated peptidyl-puromycins have an average molecular weight of 19 kDa (Fig. 4A), the average weight of polyubiquitinated proteins that accumulate in response to puromycin treatment is ∼190 kDa (Fig. 8). This suggests that the peptidyl-puromycins we observed were not directly polyubiquitinated, raising the possibility that the prematurely terminated products were degraded by the 20S proteasome via a ubiquitin-independent mechanism. This is consistent with the proposal that the 20S proteasome degrades severely misfolded RDPs [26].

Because puromycin is a premature chain terminator, increasing concentrations of puromycin are predicted to produce progressively shorter polypeptides. For the most part, we observed this relationship to be true (Fig. 2), however a subset of polypeptides approximately 14–17 kDa in size (Region 2) was unaffected by varying puromycin concentrations. We speculate that this size bias reflects the fact that, after the puromycin-mediated discharge of elongating ribosomes, the remaining initiating ribosomes (unaffected by puromycin) are clustered at the 5′ end of ORFs. Subsequent puromycin-induced termination events would then be predicted to cluster relatively 5′ along the ORFs, yielding peptidyl-puromycins of the more narrow size range observed. A corollary of this model is our speculation that two distinct populations of truncated polypeptides are sequentially produced following puromycin treatment. The first population is more heterogeneous, containing peptidyl-puromycins discharged from ribosomes distributed across the entire length of mRNAs. The second, more homogenous population contains peptidyl-puromycins discharged from ribosomes relatively closer to the 5′ end of ORFs. The potential importance of substrate heterogeneity in RDP metabolism and antigen presentation are themes discussed in more detail below.

We observe the degradation of 20% of newly synthesized polypeptides (Fig. 3B) and peptidyl-puromycins (Fig. 4B) in cells treated with proteasome inhibitors. This degradation predominantly affects low molecular weight polypeptides (Fig. 3D, MG132-treated samples). One limitation of using proteasome inhibitors is that they do not completely inactivate the proteasome, with the residual proteasomal activity showing altered substrate specificity [34]. It is therefore formally possible that the degradation we observe in the presence of MG132 represents residual proteasomal activity that is selective for small polypeptides and is activated under conditions of high substrate load. An alternative model is that this degradation is mediated by a non-proteasomal protease, such as insulin-degrading enzyme, nardilysin or a yet unidentified cytosolic protease that is selective for small or prematurely terminated polypeptides (reviewed in [35]). Indeed, under conditions of proteasomal inhibition, peptides presented by HLA-B27 molecules have been reported to derive primarily from basic proteins ranging from 6–16.5 kDa in size [36]. These observations support the existence of a yet unidentified cytosolic proteolytic system or residual proteasomal activity that is selective for small proteins. Alternatively, cleavage by non-proteasomal proteases may require the altered activity of the MG132-bound proteasome, depending on the substrate. Because MG132 is rapidly reversible and its effects on live cells have been extensively characterized, it is the most frequently utilized proteasome inhibitor in studies of RDPs. Thus, using MG132 allows us to directly compare our results to previous findings. Notably, MG132 is also a potent inhibitor of thiol proteases [21]. It will therefore be of interest to extend our findings with MG132 to other proteasome inhibitors, such as lactacystin, which is an irreversible, but more specific proteasome inhibitor. This will help clarify whether the MG132-independent degradation reported here is an effect specific to MG132 or truly represents a proteasome-independent RDP pathway. Distinguishing between these models will be important for understanding the mechanisms by which small prematurely terminated polypeptides are degraded.

Because RDPs are a source of substrates for antigenic MHC I peptides [1], [3], [4], we investigated the effects of inducing premature translational termination on MHC I presentation. We identify two contrasting experimental conditions under which low levels of prematurely terminated polypeptides correlate with increased antigen presentation. In the first scenario, short treatment with 20 µM puromycin produces a substantial, transient discharge of prematurely terminated polypeptides (Figs. 1 and 2) targeted to the RDP pathway (Figs. 3 and 4). This discharge of prematurely terminated products correlates with a significant and similarly transient increase in the rate of K^b^-SIINFEKL export at 60 min (Fig. 6C). In the second scenario, treatment with 2 µM puromycin for 10 minutes induces a small but significant increase in protein synthesis (Fig. 1A), which we speculate is a response to low levels of misfolded polypeptides [29]. The low levels of truncated polypeptides produced are likely obscured by the profile of full length proteins seen on phosphorimaging (Fig. 1B). It is therefore unsurprising that the positive effect of 2 µM puromycin on MHC I presentation is not seen until these truncated polypeptides have been continuously produced for 3 or 4 hours (Figs. 6E and 6G, respectively).

At first glance, the absolute increase in MHC class I presentation stimulated by puromycin appears small (∼6% in Figs. 6C and 6F, ∼11% in Fig. 6E). To give these results some context, in a previous peptide stripping study, antigenic substrates were allowed to accumulate in cells during two hours of complete proteasomal inhibition. Degradation of the accumulated substrates led to a 20% increase in K^b^ presentation after 3.5 hours of recovery [4]. Taking 20% as an upper limit, the puromycin-stimulated increases in MHC class I presentation we observe after 1 hour of recovery are relatively substantial.

A limitation of this study is the use of the model murine MHC class I-peptide complex, K^b^-SIINFEKL, to measure antigen presentation in human cells. The advantages of using K^b^-SIINFEKL include the robust presentation of K^b^-SIINFEKL complexes, even in heterologous cells, and the widespread availability of well-characterized reagents to measure K^b^-SIINFEKL expression, particularly for studies of RDP biology [2], [4]. However, it remains to be determined the extent to which these findings are broadly applicable to other MHC class I alleles, particularly human MHC class I molecules. Nevertheless, our work represents an important first step by examining the varied effects of premature translational termination on a model MHC class I-peptide complex.

Previous studies have reported conflicting findings on the effects of puromycin on MHC I presentation [37], [38]. These studies utilized puromycin at concentrations of 2000 µM and 200 µM, respectively. It is clear from our puromycin titration studies that the production of truncated polypeptides is (expectedly) concentration-dependent, peaking at 20 µM (in 293-K^b^ cells), and that few translation products are produced at puromycin concentrations of 200 µM or higher (Figs. 1 and 2). Furthermore, MHC class I recovery exhibited both puromycin-mediated stimulation and inhibition, depending on the concentration and duration of puromycin treatment. By establishing the dynamic range of time- and concentration-dependent effects of puromycin, we were able to observe previously unappreciated complexities in the behavior of prematurely terminated polypeptides (Figs. 3 and 4) and their processing by the MHC class I pathway (Fig. 6).

Interestingly, sustained production of prematurely terminated polypeptides correlated with the inhibition of MHC I export (Figs. 6C and 6G), impaired function of the MHC I machinery (Fig. 7), and the accumulation of polyubiquitinated proteins (Fig. 8), suggestive of proteasomal saturation. Thus, at early time points over a narrow concentration range, peptidyl-puromycins are rapidly degraded substrates for the MHC class I pathway, as initially predicted. However, the inhibition of MHC I presentation by the sustained production of truncated polypeptides indicates that the relationship between RDP production and MHC I presentation is not monotonic.

Given that RDP degradation is so tightly coupled to MHC I presentation, why do rapidly degraded peptidyl-puromycins stimulate MHC I presentation under some conditions and inhibit presentation under others? Three possible reasons (which are not mutually exclusive) for this divergent behavior are (1) decreased production of full-length proteins necessary for the function of the MHC I pathway and differences in the (2) concentration and (3) composition of premature termination products, each of which we discuss below. First, the time-dependent decline in MHC I pathway function seen with puromycin treatment (Fig. 7) likely reflects the loss of full-length proteins necessary to sustain the activity of the MHC I machinery. The late inhibition of the MHC I pathway was slightly more pronounced in cells treated with 200 µM puromycin (Fig. 7B), a concentration at which few polypeptides are synthesized (Fig. 1). This suggests that the delayed impairment in MHC I presentation is due to decreased synthesis of MHC I pathway components, rather than the accumulation of proteotoxic, truncated polypeptides (which does not occur at 200 µM puromycin). We therefore speculate that the MHC I machinery can tolerate modest or short-term inhibition of protein synthesis, while prolonged interruptions in the production of native full-length proteins deprive the MHC I pathway of essential components, leading to its shutdown. This would help ensure that the MHC I pathway is only functional while there is a steady supply of RDPs from active translation; shutdown of presentation when RDPs are not being synthesized could help prevent proteins at the end of their functional lifespan (termed “retirees”) from accessing the MHC I pathway [39].

Second, different concentrations of peptidyl-puromycins and consequent flux through proteolytic pathways may be responsible for the contrasting effects on the recovery of MHC I-peptide complexes. We speculate that low concentrations of prematurely terminated polypeptides are processed into antigenic peptides via the limited excess proteasomal capacity of the cell, leading to the observed stimulation of cell surface MHC class I expression (Fig. 6). Furthermore, we suggest that proteasomal capacity is saturated by high concentrations of premature termination products following sustained treatment with 20 µM puromycin, leading to the progressive, time-dependent inhibition of MHC I presentation. Indeed, the proteostasis network lacks significant excess capacity [40], indicating that proteasomal saturation is definitely possible if RDP flux is high. Inefficient processing of premature termination products by the MHC I machinery (discussed below) would accentuate the inhibitory effects of saturating concentrations of truncated polypeptides.

Finally, different profiles of polypeptides are produced under the experimental conditions that stimulate versus inhibit MHC I presentation. Low concentrations of puromycin (which stimulate MHC I presentation) lead to the production of polypeptides of a diverse range of sizes (Fig. 2). Indeed, immediately after being added to cells, the first wave of puromycin molecules (even at high concentrations) would be predicted to interact with ribosomes distributed across the entire length of an mRNA, leading to the initial discharge of peptidyl-puromycins spanning a diverse size range. Accordingly, there is a burst in K^b^-SIINFEKL export shortly after puromycin treatment is initiated (Fig. 6C). In contrast, high concentrations of puromycin (which inhibit MHC I presentation over time) produce truncated polypeptides over a more restricted range of smaller sizes (Figs. 2 and 4A). We speculate that the contrasting sets of polypeptides produced by these experimental conditions differ in the proteolytic pathways to which they are targeted and as a result, in the extent to which the degradation products can access the MHC class I presentation machinery.

Size-dependent differences in substrate selection for degradation pathways and processing for MHC I presentation have been previously documented ([36], discussed above), although the mechanistic basis for these differences is unclear. The observed “size-dependence” likely reflects the selective targeting of polypeptides to different degradation pathways based on the extent to which they are folded, misfolded, or unfolded [26], [32], since longer nascent polypeptides tend to have more domains that have completed folding. In our studies, lower concentrations of puromycin stimulate the premature termination of a relatively higher amount of near full-length polypeptides; the finding that these puromycin conditions are associated with increased MHC I presentation is consistent with findings from other systems that newly synthesized, full-length or near full-length defective ribosomal products (DRiPs) are the most efficient source of peptides presented on MHC class I molecules [1], [2], [39]. At the other extreme, short peptides produced by premature termination should degrade in seconds, due to the activity of cytosolic peptidases [41]. Because peptide degradation is more efficient than peptide translocation into the ER, peptide-length premature termination products would be predicted to be highly inefficient sources of MHC class I peptides.

Interestingly, a recent study by Farfán-Arribas et al. showed comparable efficiencies for peptide presentation between reporter DRiPs and mature reporter proteins, in which the antigenic peptide can only be generated after post-translational intein splicing of the functional protein [42]. These findings are in accord with earlier studies of the NSe reporter showing more efficient presentation from a slowly-degraded, misfolded reporter protein than a rapidly degraded version of the reporter [2]. Our studies with puromycin highlight the heterogeneity of premature translational termination products and suggest corresponding heterogeneity in pathways for their degradation. The common theme from our studies and others is that proteasomal substrates (both RDPs and slowly-degraded proteins) exhibit wide variation in the efficiency with which they access the MHC I pathway [2], [39], [42]. This variable efficiency has been attributed, in part, to the compartmentalization of MHC class I substrate processing, which selectively channels peptides derived from newly synthesized polypeptides for efficient presentation [43]. The basis for this compartmentalization remains mysterious (although see the studies in [44] for the provocative possibility of intranuclear translation). Therefore, identifying the distinctive features of these proteolytic pathways, the basis for their substrate selectivity, and the mechanisms that govern their access to the MHC I machinery are important future avenues for making sense of the complex calculus behind RDP metabolism.
